# Identity-by-descent analysis of CMTX3 links three families through a common founder

**DOI:** 10.1038/s10038-022-01078-1

**Published:** 2022-09-13

**Authors:** Lyndal Henden, Bianca R. Grosz, Melina Ellis, Garth A. Nicholson, Marina Kennerson, Kelly L. Williams

**Affiliations:** 1 Macquarie University Centre for Motor Neuron Disease Research, Faculty of Medicine, Health and Human Sciences, Macquarie University, Sydney, NSW Australia; 2 Northcott Neuroscience Laboratory, ANZAC Research Institute SLHD, Concord, NSW Australia; 3 Molecular Medicine Laboratory, Concord Hospital, Sydney, NSW Australia; 4 Sydney Medical School, University of Sydney, Sydney, NSW Australia

**Keywords:** Neuromuscular disease, Genetics research

## Abstract

A large 78 kb insertion from chromosome 8q24.3 into Xq27.1 was identified as the cause of CMTX3 in three families of European descent from Australia (CMT193, CMT180) and New Zealand/United Kingdom (CMT623). Using the relatedness tool XIBD to perform genome-wide identity-by-descent (IBD) analysis on 16 affected individuals from the three families demonstrated they all share the CMTX3 disease locus identical-by-descent, confirming the mutation arose in a common ancestor. Relationship estimation from IBD segment data has genetically linked all three families through 6th and 7th degree relatives.

## Introduction

X-linked Charcot-Marie-Tooth type 3 (CMTX3) disease is an inherited motor and sensory neuropathy characterised by progressive damage to peripheral nerves. Linkage mapping and whole-genome sequencing of two CMTX3 families (CMT193 from the United Kingdom/New Zealand, and CMT623 from Australia) identified the cause of disease as a 78 kb 8q24.3 insertion at chromosome Xq27.1 [1]. A founder effect has been proposed for both families who share an identical microsatellite marker-based haplotype over the CMTX3 locus, although these families were unable to be connected through genealogy and historical pedigrees. A third family (CMT180 from Australia) was subsequently found to harbour an identical 8q24.3 insertion at Xq27.1.

To investigate whether a founder effect exists in all three families, we proposed identity-by-descent (IBD) mapping. Unlike microsatellite marker-based haplotype analysis, which determines if alleles have the same nucleotide sequence (identical-by-state, IBS), IBD mapping uses single nucleotide polymorphism (SNP) data to infer chromosomal segments that have been inherited from a recent common ancestor [2]. IBD mapping allows investigators to determine the degree of relationship between pairs of individuals and is a more powerful strategy to uncover shared ancestry and founder effects compared to IBS analysis.

IBD mapping has recently been used to confirm founder effects in more than 20 families with amyotrophic lateral sclerosis with identical gene mutations [3], and genetically link four families with familial adult myoclonic epilepsy [4]. Here, we perform IBD mapping of the three CMTX3 families to: (1) confirm a founder effect in all three families, and (2) determine the closest degrees of relatedness that link the three families.

## Material and methods

SNP microarray data was obtained for 16 individuals with the chromosome 8 insertion at chromosome Xq27.1 from three CMTX3 families, genotyped on the Illumina Infinium Global Screening Array v3 and v2. An Australian control cohort of 624 samples was used as a reference population to calculate linkage disequilibrium (LD) and population-specific minor allele frequency (MAF) statistics. CMTX3 microarray data was merged with the control cohort to extract biallelic SNPs common to both cohorts. SNPs in high LD (PLINK squared correlation > 0.8 [5]) or with MAF < 0.01 were removed, resulting in 117,547 SNPs for analysis. A principal component analysis was performed against the combined HapMap Phase II and III cohorts using PLINK that showed all CMTX3 individuals and Australian control samples were of European descent. Pairwise IBD analysis was performed on the 16 CMTX3 individuals using XIBD software [6]. Shared IBD segments overlapping the disease locus at Xq27.1 were extracted to determine if an ancestral haplotype was common to all families, indicative of a founder effect. For each pair of individuals, the degree of relationship was estimated using the total fraction of genome inferred IBD as in KING [7] and reported for relatives up to 7th degree as this is the accuracy limit of the methodology [3].

## Results

**Fig. 1:**
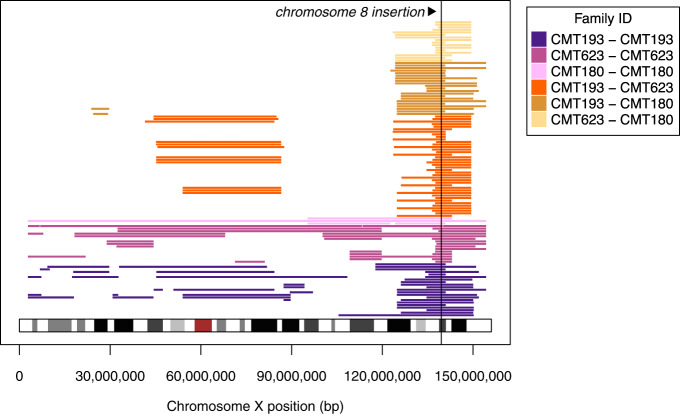
Identity-by-decent analysis of CMTX3 locus confirms a common founder. Each horizontal coloured line represents an IBD segment inferred between a unique pair of individuals either within, or between, CMTX3 families. The disease locus (chromosome 8q24.3 insertion into Xq27.1) is indicated in black. IBD segments have been coloured according to whether both individuals within a pair belong to the same family; or whether they belong to different families. For example, yellow lines at the top of the figure represent IBD segments inferred using XIBD between individuals in family CMT623 and individuals in family CMT180

**Fig. 2:**
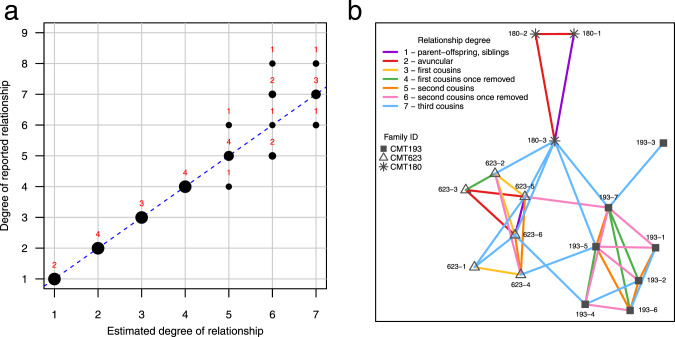
Identity-by-descent analysis confirms the degree of relationship between individuals within each family and uncovers genetic links between families. **a** IBD analysis confirms the degree of relationship to within one degree of the reported relationship as per historical pedigrees for 29/30 within-family pairs who were estimated as 7th degree relatives or closer. 1/30 pair was estimated as 6th degree relatives (second cousins once removed) when they were reported in the historical pedigree as being 8th degree relatives (third cousins once removed). Relationship estimates were only included for relatives up to 7th degree as this is the accuracy limit of the methodology [3]. The size of the circles is proportional to the percentage of pairs whose estimated degree of relationship matches that reported in the pedigree. The number of pairs estimated at each degree is labelled above the corresponding circle. Circles that fall on the dotted line, y = x, indicate concordance between the estimated degree of relationship and that reported in the historical pedigree. **b** IBD and network analysis uncovers the genetic links between families and confirms the degree of relationship between individuals within families. Each node represents an individual with a unique shape per family. Nodes are labelled to include the family ID and individual ID, for example 180-1 is individual 1 from family CMT180. A line is drawn between two individuals if they were estimated as 7th degree relatives or closer, with line colours representing different degrees of relationships

A 4.42 cM region shared IBD over the Xq27.1 disease locus was identified between all affected individuals confirming a founder effect (Fig. 1). A 53-SNP founder haplotype was extracted over the shared IBD region (chrX:138,429,585-140,025,086) and is provided in the Supplementary Data. We compared the relationships estimated from the genomic data with the relationships reported in the historical pedigrees for all pairs of individuals estimated as 7th degree relatives or closer. We confirmed 29/30 within-family relationships to within one degree of the reported relationship while estimating one within-family relationship more closely than was reported (Fig. 2a). We identified eight relationships between individuals from different families, comprising one 6th degree relative (second cousin once removed) and seven 7th degree relatives (third cousins) (Fig. 2b), thereby genetically linking these three CMTX3 families.

## Discussion

In the absence of large historical pedigrees, genetic data can be successfully used to uncover cryptic relatedness between families and confirm founder effects. Using genome-wide genotype data coupled with IBD algorithms, we have determined that the 78 kb 8q24.3 insertion at chromosome Xq27.1 arose in a common ancestor of three CMTX3 families. We genetically linked the three families by 6th and 7th degree relatives (second cousins once removed and third cousins respectively) and identified a 4.42 cM region shared identical by descent over Xq27.1 in all 16 affected individuals. Confirming a founder event in these families is unsurprising, as such a large DNA rearrangement is unlikely to have arisen independently in three families, especially considering the three families originate from either the United Kingdom, New Zealand or Australia; countries with a common largely Anglo-Celtic heritage.

More than 80 genes have been implicated in CMT to date yet ~20% of CMT cases have no known genetic cause of disease [1, 8]. This is in part due to the clinical and genetic heterogeneity of CMT, the rarity of disease, and limitations in gene discovery pipelines to uncover complex genetic variants, such as structural rearrangements, as opposed to more readily identified missense variants and small insertions and deletions [8]. These challenges are also observed in other neurological and neurodegenerative disorders, including amyotrophic lateral sclerosis, autism spectrum disorder, epilepsy encephalopathy, and intellectual disability, where a genetic cause of disease remains unknown in a large fraction of cases despite heredity studies implicating a larger genetic contribution to disease [9–12].

Linkage analysis and genome-wide association studies combined with next-generation sequencing have been successful at uncovering genetic variants implicated in these disorders. However, these methodologies have exhausted highly penetrant multi-generational families and large case-control cohorts, with alternative techniques required to solve more challenging low-penetrant families and disorders caused by rare variants. IBD mapping represents a complementary strategy that is appropriate for both familial and sporadic disease cohorts of all sizes as it can uncover cryptic relatedness to create extended families, and subsequently implicate candidate disease loci for gene discovery efforts. Here we show in CMTX3, where the causal variant was known, IBD mapping successfully confirmed a founder effect. In individuals with no known genetic cause of disease, IBD mapping can implicate new genetic loci and represents a promising technique to shed light on the missing heritability in CMT and other neurological and neurodegenerative disorders.

## Supplementary information

Dataset 1

## Data Availability

The datasets generated and/or analysed during the current study are available from the corresponding author on reasonable request.
